# Supplementary material for: Adaptive evolution of Candida maltosa improves the bioconversion of depolymerized plastic feedstock by targeting biosurfactant production
Source: Appl Environ Microbiol. 2026 Mar 4;92(4):e02056-25. doi: 10.1128/aem.02056-25 (PMC13101509; doi:10.1128/aem.02056-25)
Supplement: Supplemental material — Fig. S1 and S2; Table S1. [file aem.02056-25-s0001.docx]

**ADAPTIVE EVOLUTION OF *CANDIDA MALTOSA* IMPROVES THE BIOCONVERSION OF DEPOLYMERIZED PLASTIC FEEDSTOCK BY TARGETING BIOSURFACTANT PRODUCTION**

Efrain Rodriguez-Ocasio^1^, Kimia Noroozi^1^, Ammara Khalid^1^, Jessica Brown^2^, Robert C. Brown^1,2,3^, Mark A. Blenner^4^ and Laura R. Jarboe^1^

^1^ Department of Chemical and Biological Engineering, Iowa State University, Ames, IA, 50011, USA

^2^ Department of Mechanical Engineering, Iowa State University, Ames, IA, 50011, USA

^3^ Bioeconomy Institute, Iowa State University, Ames, IA, 50011, United States

^4^ Department of Chemical & Biomolecular Engineering, University of Delaware, Newark, DE, 19716, USA

**Supplemental Materials**

**Figure S1.** TOD_HDPE shown floating at the top of the aqueous phase post culture.


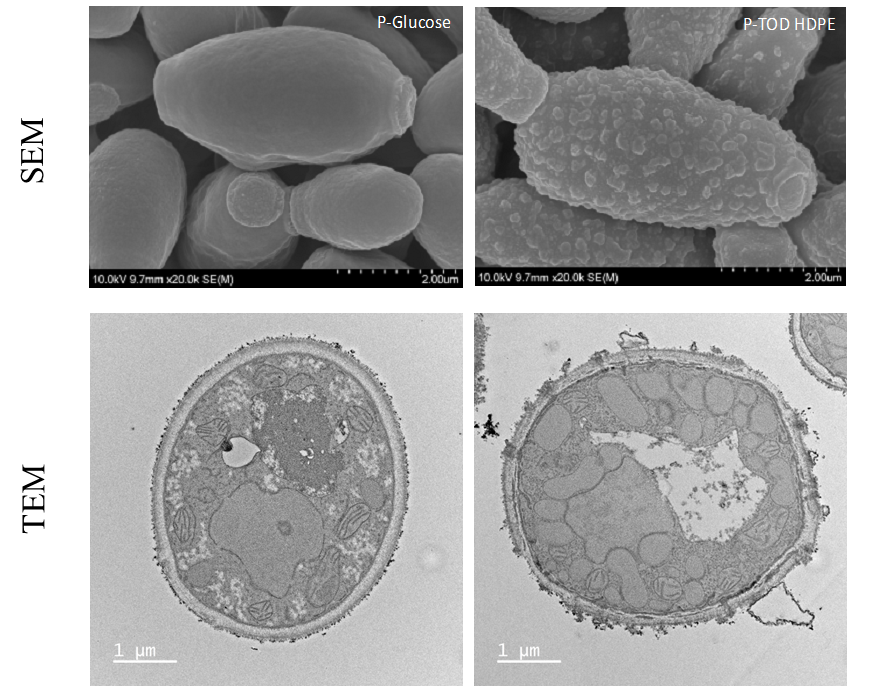


**Figure S2.** SEM and cross-sectional TEM micrographs of *Candida maltosa* show proliferation canals and intracellular compartments, including vacuoles and peroxisomes, induced by the presence of TOD_HDPE.

**Table S1.** Composition of TOD_HDPE/PP, a feedstock produced by mixing HDPE and PP plastics at a 1:1 ratio before depolymerization in the TOD reactor. Identification and quantification of the largest 30 peaks on GC-MS/FID measured of the GC-detectable fraction; the products are a mixture of alcohols, aldehydes, alkanes, alkenes, and alkadienes.

| **Compound** | **Concentration (wt.%)** |
| --- | --- |
| *Alcohol* | |
| 2-nonen-1-ol | 0.32 |
| 1-octanol, 2-butyl | 1.04 |
| 2-tridecen-1-ol | 0.40 |
| 18-nonadecen-1-ol | 0.58 |
| 1-heneicosanol | 1.38 |
| behenic alcohol | 1.52 |
| 1-heneicosanol | 1.55 |
| n-tetracosanol-1 | 0.91 |
| Octacosanol | 1.58 |
| *Aldehyde* | |
| 10-undecen-1-al, 2-methyl- | 0.35 |
| 10-undecenal | 0.34 |
| *Alkane* | |
| dodecane | 0.91 |
| hexadecane | 0.69 |
| 13-oxabicyclotridecane | 0.46 |
| 2-methyltetracosane | 0.64 |
| octadecane | 0.49 |
| eicosane | 0.54 |
| heptacosane | 0.73 |
| *Alkene* |  |
| 1-dodecene | 1.64 |
| 1-tridecene | 3.26 |
| 1-tetradecene | 2.95 |
| 1-pentadecene | 2.43 |
| cetene | 1.81 |
| 1-heptadecene | 1.50 |
| 1-nonadecene | 1.21 |
| 5-eicosene | 1.23 |
| *Alkadiene* | |
| 1,11-dodecadiene | 2.72 |
| 1,12-tridecadiene | 1.34 |
| 1,15-hexadecadiene | 0.57 |
| 1,19-eicosadiene | 0.56 |

**Table S2.** Proteome Analysis. List of intracellular (yeast pellet) and secreted (spent media) proteins identified from the parent and evolved strains of *Candida maltosa*. Table provided as a separate excel file.
